# Comparing Preferred and Actual Clinical Learning Environments and Perceptions of First-Year Nursing Students in Long-Term Care: A Cross-Sectional Study

**DOI:** 10.3390/ijerph20054500

**Published:** 2023-03-03

**Authors:** Míriam Rodríguez-Monforte, Sofía Berlanga-Fernández, Rosa Rifà-Ros, Anna Martín-Arribas, Cristina Olivé-Adrados, Rosa Villafáfila-Ferrero, Rosa M. Pérez-Cañaveras, María Flores Vizcaya-Moreno

**Affiliations:** 1 Global Research on Wellbeing (GRoW) Research Group, Blanquerna School of Health Sciences, Universitat Ramon Llull, Padilla, 326-332, 08025 Barcelona, Spain; 2 Unitat Docent Multiprofessional Atenció Familiar i Comunitària Costa Ponent, Bellaterra, 41, 08908 Barcelona, Spain; 3 GHenderS Research Group, Blanquerna School of Health Sciences, Universitat Ramon Llull, Padilla, 326-332, 08025 Barcelona, Spain; 4 Clinical Nursing Research Group, Department of Nursing, Faculty of Health Sciences, University of Alicante, 03690 San Vicente del Raspeig, Spain

**Keywords:** nurse education, clinical learning environment, clinical learning environment inventory, nursing students

## Abstract

The clinical learning environment, which includes the culture of clinical units, the mentoring process, and the different health organizations, influences the learning process of nursing students. However, scarce literature has been published on the impact of the clinical learning environment on first-year nursing students in long-term care. We aimed to assess first-year nursing students ‘preferred’ and ‘actual’ clinical learning environments when conducting their first placements in nursing homes within an innovative placement model that comprised the active participation of academic mentors. The validated Spanish version of the Clinical Learning Environment Inventory (CLEI) instrument was used in our study, and 99 first-year nursing students participated. The highest mean scores for the CLEI-Actual were found for the Satisfaction (22.7) and Involvement scales (19.09). The lowest mean scores were found for the Personalization (17) and Individualization (17.27) scales. The multiple correlation (R) between the Satisfaction and the other CLEI scales was 0.61 (*p* > 0.001), which means that in this study the association between student satisfaction and their perception of the clinical learning environment was strong. First-year students conducting their first clinical placements in nursing homes can have a positive learning experience considering a well-designed and organized pedagogical strategy, including constant support and feedback from academic and clinical mentors.

## 1. Introduction

The Bachelor’s Degree in Nursing curriculum includes two main learning scenarios: academic and clinical. In the European context, since the approval of the Bologna Declaration [1], the designation of an even number of learning hours between theory and practice (2300 h each) [2] has highlighted the importance of the clinical setting where students conduct clinical placements in contact with a diversity of contexts and actors: nursing professionals, nursing supervisors, other health team members, patients, and the physical context where placements are conducted (theatres, ICU, wards, etc.).

In this sense, clinical learning environments (CLEs) can be defined as multidimensional entities that directly affect the outcomes of students’ clinical placements [3], with different forces and elements or attributes that may include structure, organization, and attitudes [4], that is to say, the physical space, psychosocial and interaction factors, organizational culture, and teaching and learning components [5].

The CLE can strongly influence nursing students, not only concerning their learning process but also the sense of preparation for nursing practice, satisfaction with the nursing profession, professional identity, and the intention to remain as future professionals [5]. In 1987, Moos claimed that social climate can strongly influence people in a particular setting, affecting each person’s behavior, feelings, and growth [6]. Research also shows that students consider their CLE and mentoring more highly when a skilled, specific mentor is assigned to them; learning goals are discussed; a final assessment is carried out; and preclinical teaching supports learning in the clinical setting [7]. Consequently, providing a positive CLE for nursing students must be a foreseen objective for academic and clinical settings.

Different clinical settings might determine different CLEs. The literature shows that it is crucial to assess the perceptions of nursing students in a variety of settings in order to understand and predict the elements that can act as barriers and facilitators for achieving an optimal CLE [8,9]. In the nursing curriculum, hospitals are the most common settings where placements are conducted. Consequently, a wide range of literature on the CLE of hospitalization and critical care units has been produced, yielding positive results when good supervision from the clinical mentors was provided [10,11,12]. Other clinical learning spaces exist in the nursing curriculum, such as primary care or long-term care (LTC) settings, which can be of great importance for future professionals, considering the present and future population profiles [9,13]. However, less literature focuses on the CLE of these clinical settings. Some authors describe that positive learning experiences in nursing homes may increase students’ professional development through the achievement of basic nursing skills and motivate them to choose this field for their future career path [14,15].

Additionally, the time when the clinical placements take place must also be considered. The impact and outcomes of the CLE on a first-year nursing student might strongly differ from those on a final-year student. First-year nursing students face a unique challenge in their first clinical placements due to the fact of their lack of experience and a diversity of expectations of the clinical reality and of themselves that range from a very positive vision of their preparedness and an easy future clinical experience supported by skilled mentors to a negative vision of their readiness for their placement and fear of their ability to cope with all the challenges of the clinical setting [10].

The clinical mentor is a well-defined figure whose influence during clinical learning has been labeled as crucial for students [16]. Additionally, during their clinical placements, students have another reference figure: the academic mentor. In the Spanish context, this figure plays a more secondary role serving as an occasional help for the students and clinical mentors. However, the role of academic mentors can have a lot of potential, especially in students’ initial placements. Academic mentors can have access to more information concerning the students enrolled in Bachelor’s Degree in Nursing studies, and they can also ensure the possibility of follow-up on the students’ progression. Moreover, the pedagogical background of academic mentors can be more solid [17].

The assessment of the initial expectations of nursing students concerning the CLE they will encounter, especially during their first clinical placement, is critical, and little evidence has been developed around the topic [18]. Moreover, and considering the vulnerability that this first placement period represents in their learning process, the comparison between expectations versus reality can help the academic and clinical mentors to understand and reflect upon the elements that can facilitate the learning process, also considering the impact that the first clinical placements have on the intention to remain in the profession and these future professionals’ appreciation of the discipline [19].

In light of the above, the aim of our study was to assess first-year nursing students’ preferred and actual clinical learning environment for their first clinical placements in LTC within a new internship framework, including close pedagogical support by academic mentors.

## 2. Materials and Methods

### 2.1. Design

A cross-sectional survey design using a standardized self-report scale was developed. The STROBE Checklist was followed for the development and reporting of our study [20].

### 2.2. Participants, Settings, and Procedure

All first-year nursing students of the Blanquerna School of Health Sciences in Barcelona, Spain, in the 2022–2023 academic year (*n* = 154), were asked to participate in the study by responding to a self-report scale about the CLE before and after their first clinical placement experience. In Spain, the nursing degree has a duration of four years. The study was presented to the students in early October 2022, just after the presentation of their first clinical placements period. Any questions or doubts were solved during the session.

The first Clinical Learning Environment Inventory (CLEI) survey, which includes information about the student’s ‘preferred’ learning environment, was conducted in early October 2022 [3]. The students’ first clinical placements started in mid-October 2022 and ended at the beginning of December 2022. They involved a two-week placement in LTC, specifically in a nursing home. During their placements, the students also carry out clinical simulations guided by the same academic mentor as they have on their placements. On each day of their placements, the students were accompanied by an academic mentor who served as an active support figure for them.

Each academic mentor had a group of approximately 10 to 12 students. Each day started with a briefing session of 30 min in which the objectives of the day, expectations, fears, and any other topic the group decided to highlight were shared. Afterwards, the academic mentor accompanied the students to each of the wards where they were assigned in the nursing home and, with the clinical mentor, shared the expectations, objectives, and tasks that the students aimed to perform. In the middle of the day, the academic mentor conducted a brief follow-up on the students’ performance by asking their clinical mentors and the students themselves if there were any issues they wished to comment on. At the end of the shift, the academic mentor met for a one-hour debriefing session with the students, where they shared the experiences of their working day with the group, as well as any critical incidents, which were analyzed using Gibbs’s reflective model [21]. In December, when the placements were about to finish, a new survey was conducted in order to gather information about the ‘actual’ CLE. Both surveys (Actual and Preferred versions of the CLEI) included questions regarding the students’ sociodemographic characteristics.

The students took 10 to 15 min to complete the Preferred and Actual versions of the CLEI, which were completed on a voluntary basis. The voluntary nature of the study information was also stipulated at the beginning of the online survey. The results of the questionnaire, which were anonymously gathered through the online platform Google Forms, were presented to the different groups of students one week after the placements had finished.

Convenience sampling was used to source participants, considering the following criteria: (a) be a first-year student in our faculty, (b) be able to provide their consent to take part in the study, and (c) be in the first year as a nursing student and not between years.

The appropriate statistical sample size, determined through the Netquest sample-size calculator, reflects that a minimum response rate of 98 would be needed to ensure a 95% confidence level and an error margin of 6%.

### 2.3. Instrument

Chan developed the Clinical Learning Environment Inventory (CLEI) to measure the clinical learning environment while nursing students are completing education placements in the field [3,22,23]. The CLEI assesses students’ perceptions of the psychosocial characteristics of the clinical learning environment, where students, clients, clinicians, clinical fieldwork supervisors, and other health providers work, interact, and coexist. An important feature of this framework, and one which has been applied to many recent classroom environment instruments, is that it has two distinct forms: one measuring students’ perceptions of the ‘actual’ environment, while the other measures the environment ‘ideally liked or preferred’ by students [3,22].

This 42-item question inventory is structured into six scales (Table 1) with seven items evaluated by a four-point Likert scale (strongly agree = 4; strongly disagree = 1). The inventory includes items that score in reverse in the ‘actual’ (2, 6, 14, 21, 22, 27, 41, and 42) and ‘preferred’ (2, 5, 6, 9, 11, 14, 16, 25, 26, 27, 29, 31, 36, 37, 38, 41, and 42) forms. The score for each scale ranges from 7 to 28.

The internal consistency of the CLEI had been confirmed in previous studies worldwide. For example, the Cronbach’s alpha coefficients ranged from 0.73 to 0.84 (Actual form) and 0.68 to 0.80 (Preferred form) in Australia [3,23]; from 0.50 to 0.80 (Actual form) and 0.51 to 0.76 (Preferred form) in Hong Kong [24]; from 0.86 to 0.89 (Actual form) and 0.73 to 0.76 (Preferred form) in Spain [25]; from 0.47 to 0.74 (Actual and Preferred forms) in Italy [12]; from 0.43 to 0.86 (Actual and Preferred forms) in Norway [26]; from 0.55 to 0.76 (Actual form) and 0.58 to 0.77 (Preferred form) in Greece [27]; from 0.61 to 0.9 (Actual form and Preferred forms) in Ireland [28]; from 0.73 to 0.82 (Actual and Preferred forms) in Egypt [29]; and from 0.73 to 0.84 (Actual form) and 0.47 to 0.92 (Preferred form) in Singapore [30].

The English version of the CLEI was translated into Spanish using the modified direct translation method [31] after obtaining permission from the questionnaire’s author [3]. An expert panel of two nurse teachers translated the original instrument individually and then discussed their work together, analyzing the different proposals until they reached a consensus on each item to obtain a single version in Spanish of the different items. Subsequently, a native translator translated the Spanish version back into English. Lastly, the cross-cultural equivalence of the final Spanish version was verified. After piloting both forms of the question inventory in a sample of 48 nursing students [32], the Spanish CLEI-Actual and CLEI-Preferred questionnaires were administered to 308 nursing students [25]. No problems were detected, and no modifications were required.

### 2.4. Statistical Analysis

The IBM-SPSS Version 26.0 (SPSS) was used for data entry, storage, and retrieval [33]. The student participant responses to the CLEI were tabulated, and descriptive statistics, such as measures of central tendency and measures of variance, were calculated. The reliability of the instrument was estimated using Cronbach’s alpha coefficients that examine the internal consistency of the Spanish version of the CLEI. The pairwise t-test was utilized to determine the difference between the Actual and Preferred CLEIs. Pearson’s (r) correlation coefficient and the multiple linear regression model were used to explore possible relations between nursing students’ Actual Satisfaction and the other scales of the CLEI-Actual. The CLEI Actual-*Satisfaction* scale has been employed as an outcome measure in previous studies [3,27]. The results were considered statistically significant if the *p*-value was <0.05.

### 2.5. Ethical Considerations

The Ethics Committee of the Blanquerna School of Health Sciences granted its approval for the study (CER-FCSB-2020-06-03). The participants received information about the study with the invitation to participate, and their voluntary participation was interpreted as their informed consent to participate. Data pseudonymization was used to maintain anonymity and confidentiality during all data collection and analysis procedures. The participants were provided, in a clear and comprehensible way, adequate, truthful, and timely information about their rights and the characteristics of the research. The information was processed in accordance with Organic Law 3/2018, of December 5, on the Protection of Personal Data and Guarantee of Digital Rights [34].

## 3. Results

### 3.1. Sample

A total of 154 first-year nursing students on the undergraduate programme were invited to participate in the study, with a response rate of 64.3%. All of them spent their clinical placement in a long-term residential care setting. The final consecutive sample consisted of 99 students, 79 (79.8%) females and 20 (20.2%) males. They were aged between 17 and 42 years (mean = 21.05, SD = 5.44). Only 34 (34.3%) of the participants had previous experience as health workers (health technicians or administrative staff).

### 3.2. Reliability of the Actual and Preferred CLEI

In this study, Cronbach’s alpha was 0.78 for CLEI-Actual (ranging from 0.75 to 0.81) and 0.59 for CLEI-Preferred (ranging from 0.54 to 0.61).

### 3.3. Students’ Perceptions of the Actual Clinical Learning Environment

Most participants indicated that they strongly agreed with the level of *Satisfaction* (71.7%) and agreed with the rest of the CLEI scales. Higher levels of disagreement were found for the *Personalization* (11.2%) and *Individualization* (11.1%) scales. None of the participants strongly disagreed with the CLEI-Actual items (Table 2).

### 3.4. Differences between Students’ Preferred and Actual CLEI Scores

The highest mean scores for the CLEI-Actual were found for the Satisfaction (22.7) and Involvement scales (19.09) (Table 3). The lowest mean scores were found for the Personalization (17) and Individualization (17.27) scales. The highest mean scores for the CLEI-Preferred were found for the Personalization (24.4) and Satisfaction scales (23.22). The lowest mean score was found for the Individualization (18.66) (Table 3).

The pairwise t-test utilized to determine the differences between the Actual and Preferred CLEI scores showed statistically significant differences for Personalization, Task orientation, Innovation, and Individualization scales (Table 3). The difference between the scale means ranged between 0.28 and 7.4. The most significant difference was found in the Personalization scale and the smallest in the Involvement scale.

### 3.5. Relationship between Students’ Level of Satisfaction and Their Perception of the Actual Clinical Learning Environment

The *Satisfaction* scale was employed as an outcome measure of the CLEI, exploring its relationship to environmental and psychosocial characteristics represented by the other scales of the CLEI (Personalization, Involvement, Task orientation, Innovation, and Individualization). The data analysis indicated a statistically significant positive correlation between the Satisfaction scale and the other scales (Table 4). The Pearson correlation coefficients ranged from 0.56 (*p* < 0.001) for Task orientation to 0.21 (*p* = 0.03) for Involvement.

Furthermore, the multiple linear regression model was used to explore the possible relationships between the Satisfaction (dependent variable) and the rest of the CLEI scales (independent variables) (Table 5). The data analysis showed a statistically significant positive relationship between the Satisfaction and Task orientation scales (β = 0.48, *p* < 0.001), which suggests that the extent to which ward activities are clear and well organized is closely linked with the students’ satisfaction during their clinical learning.

The multiple correlations (R) between the Satisfaction and the other CLEI scales was 0.61 (*p* > 0.001) (Table 5), which means that the association between student satisfaction and their perception of the clinical learning environment was strong in this study. The independent variables explain 37% of the variability of the dependent variables in this model. In this study, only Task orientation was found to be a significant predictor of first-year nursing students’ self-reported Satisfaction with the clinical learning environment.

## 4. Discussion

To the best of our knowledge, this is the first study to analyze the ‘preferred’ and ‘actual’ clinical learning environments of first-year nursing students in LTC placements framed in an innovative placement model that comprised the active participation of academic mentors.

Our results show that the students were highly satisfied with their clinical placements, with no significant differences between the Actual and Preferred *Satisfaction* and *Involvement* scales, which could mean that overall, they were able to enjoy and actively participate in their clinical learning process.

Little literature has been devoted to the assessment of the CLE of nursing students in LTC settings, despite studies showing the need for investment in geriatric and long-term care content in the nursing degree curriculum and that direct contact with this clinical environment and older people can improve the interest of nursing students for this future field of work [35,36,37]. Previous studies assessing the CLE were mostly conducted in hospital settings [10,11,38] and reported results that differed from the ones in our study in the sense that the overall ‘actual’ and ‘preferred’ clinical learning environments usually differ significantly from one to another [12,27,29,39,40,41], meaning that students report low satisfaction levels with their actual placements.

In LTC, Berntsen and Bjork, in 2010 and 2014, used the CLEI to evaluate first-year and last-year nursing students’ CLE in nursing homes, finding that students perceived their learning environment to be moderately positive [9,26]. Other authors have also assessed the impact of the CLE in LTC with other scales [15], also finding moderate positive results. It might appear paradoxical that in a clinical setting with worse nurse–patient ratios and a lack of resources [42,43] students rate the CLE as positive. The interpretation of this data might take into consideration various aspects.

In the case of our study, an important highlight is the innovative framework in which the placements took place, which may have helped balance the overall satisfaction of the students compared to the other scales where statistical differences existed between the CLEI-Actual and Preferred (Personalization, Individualization, and Task orientation scales).

Our model was defined by the active presence and guidance of the academic mentor for the students but also by the clinical mentors (i.e., nurses), where the goals and expectations of placements were shared daily. A study by Berntsen and Bjork also highlighted the coordination of nurses and clinical teachers as a relevant aspect that could have positively influenced their results [26].

The Innovative model described in our study was also defined by the constant use of reflective practices within a group of peers. This pedagogical strategy was highly valued by the students, who qualitatively expressed the usefulness of this tool, especially when critical incidents were analyzed. Additionally, shared reflective thinking in small groups could explain the fact that, in terms of involvement, every student had the possibility to share and reflect on the uniqueness of their own experience on each of the placement days, consolidating the sense of belonging and active participation in their placements. The students manifest that the placement did not start and end in the clinical units but in the briefing and debriefing sessions held every day with the academic mentor and their peers. Other research has described the reflective practice and peer-to-peer learning as powerful resources for the integration of knowledge in clinical practice and predictive elements for satisfactory placements [44,45]. These resources should be ideally led and conducted by clinical mentors; however, the clinical reality, especially after the COVID-19 pandemic, is even more challenging for nurses, who work with very low ratios and under great pressure in clinical settings, especially in LTC [43]. Consequently, nurses truly struggle to create these spaces for students [46].

In addition, the fact that these placements took place in parallel with clinical simulations might have also helped the students feel that they participated in and practiced the different clinical contents. Even though there is no substitute for reality [47], clinical simulation is a pedagogical tool through which students can safely practice and prepare for future challenges and reflect upon them through guided reflection and critical thinking [48,49].

Another aspect that should be considered is that the preparation of clinical mentors, in terms of pedagogical resources in Spain, is very limited, as to date no formal/official training on mentorship exists for these professionals [50]. This can have a very negative impact on students, as pedagogical learning results linked to a clear pedagogical structure and itinerary in the clinical setting are needed. The literature shows that students require constant feedback and orientation from their mentors to grow as future professionals [51,52,53]. In line with these previous ideas are the data of our study related to Personalization (17), Individualization (17.27), and Task orientation (20.22), with the results of the Actual Questionnaire suggesting that students generally seek greater interaction with clinical mentors, oriented tasks tailored to their profile, and greater structure and organization of the activities they need to perform.

Personalization was the highest-rated scale in the Preferred Questionnaire, whereas it had the lowest score in the Actual Questionnaire. Similar to our results, in other studies using the CLEI, the dimension of Personalization has been rated as the most important one for the students in the Actual scale [28,30,54] and with significant differences found between the two scales (Actual and Preferred) [3,23,27,44]. Particularly in nursing homes, Bernsten and Bjork state that high scores for Personalization may be related to the individual orientation towards students found in the supervisory system in these clinical settings [26]. We argue that, additionally, interaction with the clinical mentor needs to be seen as fundamental for nurses as an investment in the nursing profession in all clinical settings. In addition, this vision needs to be worked on through specific training, as well as compensated through career development opportunities [17].

This same proposal can be extrapolated to the dimensions of Individualization and Task orientation, where students seemed to expect to have greater interaction with clinical mentors and better organization in the performance of their duties.

In line with our results, other research has found Individualization to be rated low or very low on the Actual scale in nursing students [3,27,29,30,55] and other health disciplines [41], where students did not feel able to make their own decisions or were not able to develop their full potential.

Additionally, and inversely to our results, Task orientation has yielded positive results in the recent literature with final-year nursing students (3rd and 4th years) conducting their placements in hospitals [29,30]. These differences could be understood by the fact that our sample consisted of first-year nursing students the majority of whom had no previous experience in the clinical arena, meaning they needed a more guided path and mentorship throughout their placements as they had not developed enough skills and knowledge to start being autonomous. In this sense, differences in the dedication of mentors among the different profiles of nursing students depending on the year of the degree for which they are enrolled should be noted [56]. Smedley and Morey, back in 2010, already advised nurse educators to communicate specific assignments (as active participation will not automatically occur), discuss clear expectations, and highlight the value of feedback [53].

Innovation was also rated low, with significant differences found between the Actual and Preferred scales, meaning that innovative experiences and pedagogical resources and allocations during their clinical placements are demanded by the students. These results are also in line with previous studies [3,24,26,28,30] but need to be contextualized to current times. Innovation is challenging in academia. Universities should try to incentivize innovation by training their staff on developing new pedagogical resources, including digital tools in the academic field, but innovation should also be present in clinical placements [57]. This new trend has gained importance, especially after the COVID-19 pandemic and considering the characteristics of Generation Z nursing students: digital natives that prefer teaching methods linking mentorship learning to clinical experiences, online tutorials or videos, interactive gaming, and virtual learning environments [58]. As argued before, in the clinical setting, work pressure, poor ratios, and the lack of any pedagogical resources truly challenge the presence of innovation in the CLE. Consequently, considering this important limitation, the design of new training courses for nursing mentors needs to take innovation into account as an important element for students, and current evidence suggests that the incorporation of innovation is not a synonym for losing quality but of optimizing time and resources [13].

The low ratings on the Innovation scale could also be related to the geriatric area where the placements took place [26,35]. In the collective imagination of nursing students’, older adults are not their priority when considering the patient profile or the clinical setting in which they wish to practice, with research showing negative attitudes towards this population for many years. However, the extent to which students receive positive references in this clinical environment from their nursing lecturers and work with older adults during their placements can positively influence this view [59,60,61,62,63].

Furthermore, the multiple linear regression model used to explore the possible relationships between Satisfaction and the rest of the CLEI scales showed a statistically significant positive relationship between the Satisfaction and all the scales of the questionnaire, especially with the Task orientation scale, suggesting that the extent to which ward activities are clear and well organized is closely linked with students’ satisfaction during their clinical learning and is a significant predictor of first-year nursing students’ self-reported satisfaction with the CLE. Satisfaction has been linked to self-efficacy by previous authors [29] and with stress perceived in the CLE [64]. It is an important dimension to explore in order to understand which elements should be embedded to improve students’ clinical learning process. The orientation of the students’ tasks is closely related with the level of pedagogical competence of the clinical mentors and with the protected time to design a thoughtful itinerary for the students they will receive in their units [17].

In terms of limitations, the present study was conducted with the participation of first-year students from just one university. However, the placements were carried out in different nursing homes. The students completed a self-report questionnaire, which may influence the way questions are answered, with respondents wishing to provide socially desirable and acceptable responses. The observational design limits the possibility of establishing causation; however, the results are in line with those from previous research that used the CLEI [27,28,29,30,51,54]. The study sample was a convenience sample. The students belonged to the first year of the bachelor’s degree, and some of them, though only a low number, had a background of some clinical experience. The placements were conducted according to a very specific innovation model. Consequently, any generalization of these results should be treated with caution.

## 5. Conclusions

The results of our study support the idea that first-year students conducting their first clinical placements in nursing homes can have a positive learning experience considering a well-designed and organized pedagogical strategy, including constant support and feedback from academic and clinical mentors. The CLEI proved to be an optimal tool to assess the CLE of nursing students, with the results showing high levels of satisfaction especially influenced by clear and well-organized ward activities. Our results suggest that close collaboration between academic mentors and clinical mentors and the integration of the learning process that starts in the classroom with the clinical placement experience is a highly positive element for nursing students’ learning. This strategy may also facilitate the pending closure of the academic–practice gap.

## Figures and Tables

**Table 1:** Definition of the CLEI scales (source: Chan, 2001, p. 629 [22]).

Scales Name	Description	Items
Personalization	Emphasis on opportunities for individual students to interact with clinical teacher/clinician and on concern for student’s personal welfare.	1, 7, 13, 19, 25, 31, 37
Involvement	Extent to which students participate actively and attentively in hospital ward activities.	2, 8, 14, 20, 26, 32, 38
Task orientation	Extent to which ward activities are clear and well organized.	4, 10, 16, 22, 28, 34, 40
Innovation	Extent to which clinical teacher/clinician plans new, interesting, and productive ward experiences, teaching techniques, learning activities, and patient allocations.	5, 11, 17, 23, 29, 35, 41
Individualization	Extent to which students are allowed to make decisions and are treated differentially according to ability or interest.	6, 12, 18, 24, 30, 36, 42
Satisfaction	Extent of enjoyment of clinical field placement.	3, 9, 15, 21, 27, 33, 39

**Table 2:** Frequency score of the CLEI-Actual (*n* = 99).

CLEI Scale	Actual				
	Mean (SD) *	Strongly Agree **	Agree **	Disagree **	Strongly Disagree **
Personalization	17 (1.95)	-	88 (88.8%)	11 (11.2%)	-
Involvement	21 (2.13)	38 (38.5%)	60 (60.5%)	1 (1%)	-
Task orientation	20.22 (2.33)	34 (34.4%)	64 (64.6%)	1 (1%)	-
Innovation	19.09 (2.47)	15 (15.1%)	80 (80.9%)	4 (4%)	-
Individualization	17.27 (2.26)	2 (2%)	86 (86.9%)	11 (11.1%)	-
Satisfaction	22.7 (2.96)	71 (71.7%)	26 (26.3%)	2 (2%)	-

* *n* (%). ** Strongly agree = 4; strongly disagree = 0.

**Table 3:** Differences between the mean scores of the CLEI-Preferred scales and CLEI-Actual scales (*n* = 99).

CLEI Scale	Mean ± SD ^1^		Mean Difference (95% CI ^2^)	t	df	*p*-Value
	Preferred	Actual				
Personalization	24.4 ± 2.15	17 ± 1.95	7.4 (6.81–7.99)	25.08	98	<0.001
Involvement	21.28 ± 1.83	21 ± 2.13	0.28 (−0.31–0.88)	0.94	98	0.34
Task orientation	21.23 ± 1.71	20.22 ± 2.33	1.01 (0.44–1.57)	3.54	98	<0.001
Innovation	20.35 ± 2.39	19.09 ± 2.47	1.26 (0.56–1.96)	3.57	98	<0.001
Individualization	18.66 ± 2.19	17.27 ± 2.26	1.38 (0.79–1.97)	4.63	98	<0.001
Satisfaction	23.22 ± 1.64	22.7 ± 2.96	0.52 (−0.79–1.13)	1.72	98	0.08

^1^ Standard deviation. ^2^ Confidence Interval.

**Table 4:** Pearson correlation coefficients between the Satisfaction scale and the other scales of the CLEI-Actual.

CLEI Scale	Pearson Correlation Coefficient (r)	*p*-Value
Personalization	0.339	<0.001 *
Involvement	0.216	0.032 **
Task orientation	0.562	<0.001 *
Innovation	0.352	<0.001 *
Individualization	0.297	0.003 *

* Correlation is significant at the 0.01 level. ** Correlation is significant at the 0.05 level.

**Table 5:** Multiple linear regressions with the Satisfaction scale as the dependent variable and the other Actual-CLEI scales as predictors.

Independent Variables	Coefficient β (95% CI ^1^)	*p*-Value	Multiple Correlation, R	R^2^	F (Sig.)
Personalization	0.14 (−0.08–0.50)	0.15	0.61 (*p* < 0.001)	0.37	11.016 (<0.001)
Involvement	−0.03 (−0.29–0.21)	0.75			
Task orientation	0.48 (0.37–0.84)	<0.001			
Innovation	0.06 (−0.17–0.32)	0.54			
Individualization	0.14 (−0.05–0.41)	0.11			

^1^ Confidence Interval. R^2^ = R square; F = F change.

## Data Availability

The data presented in this study are available upon request from the first author.
